# Rivaroxaban-Induced Hemorrhage Associated with *ABCB1* Genetic Defect

**DOI:** 10.3389/fphar.2016.00494

**Published:** 2016-12-19

**Authors:** Kuntheavy Ing Lorenzini, Youssef Daali, Pierre Fontana, Jules Desmeules, Caroline Samer

**Affiliations:** ^1^Division of Clinical Pharmacology and Toxicology, University Hospitals of GenevaGeneva, Switzerland; ^2^Division of Angiology and Haemostasis, University Hospitals of GenevaGeneva, Switzerland

**Keywords:** direct oral anticoagulants, adverse drug reaction, genetic polymorphism, *ABCB1*, CYP3A4/5, drug-drug interaction

## Abstract

We report a patient who presented a non-ST segment elevation myocardial infarction in the context of severe normocytic hypochromic anemia related to gastrointestinal bleeding, 3 months after switching anticoagulant from the vitamin K antagonist acenocoumarol to the direct oral anticoagulant rivaroxaban. High levels of both anti-Xa activity and rivaroxaban plasma concentrations were measured despite rivaroxaban withdrawal, suggesting reduced elimination/drug clearance. Estimated half-life was 2–3 times longer than usually reported. The patient is a homozygous carrier of *ABCB1* variant alleles, which could have participated to reduced elimination of rivaroxaban. Furthermore, CYP3A4/5 phenotyping showed moderately reduced enzyme activity. Drug-drug interaction with simvastatin may have contributed to decreased rivaroxaban elimination. Although in the present case moderate acute renal failure probably played a role, more clinical data are required to elucidate the impact of *ABCB1* polymorphism on rivaroxaban pharmacokinetics and bleeding complications.

## Introduction

Rivaroxaban is a direct Factor Xa inhibitor which has been approved for specific thromboembolic disorders such as the prevention of stroke and systemic embolism in adults with non-valvular atrial fibrillation (Mueck et al., 2014). Rivaroxaban is both excreted as unchanged drug in urine and through metabolic transformation. P-glycoprotein (P-gp) and breast cancer resistance protein (BCRP), as well as cytochrome P450 (CYP) 3A4/5 have been shown to play major roles in rivaroxaban transport and metabolism, respectively (Mueck et al., 2014). The administration of strong inhibitors of CYP3A4/5 and P-gp/BCRP such as ritonavir and ketoconazole has been shown to increase rivaroxaban exposure and cases of severe bleeding have been reported (Mueck et al., 2013). However, to the best of our knowledge, no human data has described the impact of genetic polymorphism of *ABCB1* and/or *ABCG2* on the pharmacokinetics and safety of rivaroxaban. *ABCB1* and *ABCG2* genes encode for P-gp and BCRP eﬄux transporter, respectively, (Hodges et al., 2011; Giacomini et al., 2013). We report here a rivaroxaban-treated patient who presented with severe anemia related to gastrointestinal bleeding and in whom *ABCB1* genetic polymorphism and drug-drug interaction (DDI) may have been contributing factors. The patient gave his written informed consent for publication of this report.

## Case Presentation

Our patient is a 79-year-old male suffering from systolic cardiac failure (ischemic, rhythmic, and valvular) and type 2 diabetes mellitus. The patient had received rivaroxaban 20 mg q.d. since September 2015 for cardioembolic strokes and atrial fibrillation. Before the introduction of rivaroxaban, he had been treated with acenocoumarol for years. The patient was hospitalized on December 15th 2015 for non-ST segment elevation myocardial infarction (NSTEMI). At hospital admission, laboratory testing showed severe normocytic hypochromic anemia with a hemoglobin level at 70 g/l (normal range: 140–180 g/l), without hemodynamic instability. The patient received erythrocyte transfusions, which raised the hemoglobin to 105–110 g/l. Acute renal failure was also diagnosed with a CL_CR_ value at 39 ml/min using the Cockcroft–Gault equation at admission. Renal function improved at 57 ml/min 4 days later. Due to the presence of fecal occult blood on two occasions, iron loss from gastrointestinal bleeding was suspected. The colonoscopy did not show any evidence of colon injury; however, inadequate bowel preparation was highlighted by the examinator. Gastroscopy could not be performed because the patient’s comorbidities exposed him to high risks in case of general anesthesia. Rivaroxaban was stopped at admission; enoxaparin was introduced 4 days later and then switched to acenocoumarol.

The other patient medications before hospitalization were: insulin, simvastatin 40 mg q.d., levothyroxine 75 μg q.d., extended-release metoprolol 25 mg q.d., and enalapril 10 mg q.d.

## Investigations

Clinical investigations were performed to assess for causes of potential increased rivaroxaban effects at therapeutic doses. They included anti-Xa activity measurement, rivaroxaban plasma concentrations measurement, as well as *ABCB1* genotyping, and CYP3A4/5 phenotyping.

### Anti-Xa Activity

Anti-Xa activity was measured with a chromogenic assay using the DiXal^®^ kit (Hyphen Biomed, Neuville-Sur-Oise, France) and a BCS XP instrument (Siemens, Marburg, Germany). This method has a limit of detection of 10 ng/ml. No information is given by the manufacturer regarding the limit of quantification (LOQ). However, previous studies have shown a LOQ of 20–30 ng/ml (Douxfils et al., 2013). The accuracy and precision calculated from the quality controls (QCs) were 107.0 and 8.8%, respectively, (Asmis et al., 2012). An excellent correlation between this method and liquid chromatography-tandem mass spectrometry (LC-MS/MS) has been shown (Spearman correlation coefficient of 0.96) (Douxfils et al., 2013).

### Rivaroxaban Plasma Concentrations

Rivaroxaban determination was performed using a fully validated LC-MS/MS method according to guidelines of the US Food and Drug Administration and the International Conference on Harmonization. The method was accurate and precise across the dynamic range of 0.5–1000 ng/ml. The LOQ was 0.5 ng/ml. The mean precision and accuracy, calculated from the QCs, were 10.2 and 112%, respectively.

A plasma sample of 40 μl was processed by protein precipitation extraction using acetonitrile (200 μL). Separation was performed on a C18 column (50 mm × 2.1 mm ID; 2.6 μm particle size) and under gradient conditions using formic acid 10 mM in water and formic acid 10 mM in acetonitrile. Detection was by tandem-MS in positive mode using a Qtrap API 6500 from AB sciex (Ontario, Canada) using rivaroxaban-d4 as internal standard (20 ng/ml).

### *ABCB1* Genotyping

Genomic DNA was extracted from whole blood (200 μl) using the QIAamp DNA blood mini kit (QIAGEN, Hombrechtikon, Switzerland). *ABCB1* c.3435C>T and c.2677G>T polymorphisms were determined in a single multiplex PCR, with fluorescent probe melting temperature analysis on a LightCycler (Roche, Rotkreuz, Switzerland) as previously described (Ansermot et al., 2008).

### CYP3A4/5 Phenotyping

Midazolam was used as a probe to measure the joint activity of CYP3A4/5 as previously described (Bosilkovska et al., 2014). Phenotyping was performed 8 days after hospital admission with concomitant treatment of insulin, enoxaparin 60 mg b.i.d., atorvastatin 40 mg q.d. (replacing simvastatin from the day of hospital admission), esomeprazole 40 mg q.d., levothyroxine 75 μg q.d., lisinopril 10 mg q.d., extended-release metoprolol 50 mg q.d., picosulfate 5 mg q.d., and spironolactone 25 mg q.d.

## Results

Results from anti-Xa activity and rivaroxaban plasma concentrations are presented in **Table 1**. The patient was a homozygous carrier of both tested *ABCB1* variant alleles. His genotype was TT for the c.2677G>T single nucleotide polymorphism (SNP) and TT for the c.3435C>T SNP. CYP3A4/5 phenotyping showed moderately decreased enzymatic activity, with OH-midazolam/midazolam metabolic ratio of 0.31.

**Table 1:** Anti-Xa activity and rivaroxaban plasma concentrations.

Time after the lastrivaroxaban dose	Anti-Xa activity (ng/ml)	Rivaroxaban plasmaconcentrations (ng/ml)
24 h	231	not performed
48 h	110	88.3
60 h	81	not performed
72 h	66	70.5
84 h	66	not performed
96 h	34	35.6

## Discussion

We described the case of a rivaroxaban-treated patient who presented a non-ST segment elevation myocardial infarction in the context of a severe anemia probably due to a gastrointestinal bleeding, 3 months after switching anticoagulant treatment from acenocoumarol to rivaroxaban. Laboratory investigations showed high levels of anti-Xa activity and rivaroxaban plasma concentrations at trough level (24 h after the last dosing) and an unexpected delay for rivaroxaban clearance, suggesting impaired rivaroxaban elimination. Our hypothesis is that both genetic and environmental factors might have contributed to an increased susceptibility to rivaroxaban in this patient, e.g., the homozygous presence of *ABCB1* variant alleles and reduced CYP3A4/5 activity due to DDI with simvastatin. Given that more than one third of the dose is eliminated as unchanged active drug in the urine (Mueck et al., 2014), acute renal failure was probably also a contributing factor. However, renal impairment at admission was moderate (39 ml/min), not requiring dose adjustment according to the summary of product characteristics. In a physiologically based pharmacokinetic (PBPK) model simulating the combined effect of renal impairment and concomitant erythromycin (combined P-gp and moderate CYP3A4/5 inhibitor) administration on rivaroxaban pharmacokinetics, concurrent renal impairment plus erythromycin resulted in a 2.5–3 fold increase in rivaroxaban exposure in elderly (Grillo et al., 2012). However, *in vivo* data showed that the impact of concomitant renal impairment and erythromycin administration was less than expected by the PBPK model. Indeed, a clinical study showed that in subjects with moderate renal impairment receiving concomitant erythromycin, rivaroxaban AUC, and Cmax values increased by approximately 99 and 64%, as compared with subjects with normal renal function receiving rivaroxaban 10 mg alone (Moore et al., 2014).

Rivaroxaban belongs to the recently developed class of direct oral anticoagulants (DOACs). Older OACs such as vitamin K antagonists (VKAs) are characterized by extensive hepatic metabolism by CYP450, narrow therapeutic window and the need for routine coagulation monitoring for dose adjustment. Their extensive hepatic metabolism implies multiple potential DDI and an impact of CYP2C9 genetic polymorphism on their pharmacokinetics (Scaglione, 2013). DOACs are given at fixed doses without the need for routine monitoring (Mueck et al., 2014). However, these compounds are also subject to DDI due to CYP450-mediated metabolism and/or P-gp and/or BCRP-mediated transport (Scaglione, 2013). About one third of a rivaroxaban dose is renally excreted as an unchanged form (Mueck et al., 2014) via glomerular filtration and tubular secretion (Scaglione, 2013). P-gp and BCRP have been shown to be the major transporters involved in the active renal secretion of rivaroxaban (Mueck et al., 2014). The remaining part of the dose is eliminated after hepatic metabolism. CYP3A4/5 and CYP2J2 are the isoenzymes involved in rivaroxaban metabolism, while CYP-independent mechanisms are also contributing (Mueck et al., 2014).

To the best of our knowledge, no human data have yet described the impact of *CYP3A4/5* and/or *ABCB1 (MDR1)* and *ABCG2* gene polymorphism on the pharmacokinetics and safety of rivaroxaban. More than 100 single nucleotide polymorphisms (SNPs) of the *ABCB1* gene region occurring at a >5% frequency have been described. For the most common coding SNPs (c.2677G>T, c.3435C>T), allele frequencies exhibits large interethnic differences, ranging between 2 and 90% across populations and depending on the SNP (Hodges et al., 2011). These SNPs are in high linkage disequilibrium and are therefore observed most frequently as haplotypes. In the Caucasian population, the TT TT haplotype frequency ranges from 15 to 25% (Marzolini et al., 2004).

An animal study showed that Mdr1a/Mdr1b/Bcrp triple knockout mice exhibited higher rivaroxaban plasma levels than wild type mice, with a 1.7 fold increase in plasma concentration 4 h after administration (Gong et al., 2013). The observed effect was explained by decreased excretion of rivaroxaban via P-gp and/or BCRP rather than increased absorption as rivaroxaban oral bioavailability is high (80–100%; Mueck et al., 2014). *ABCB1* genotype of our patient was a homozygous carrier for both tested SNP (c.2677G>T: TT; and c.3435C>T: TT). This genotype might have contributed to the high levels of anti-Xa activity and rivaroxaban plasma concentrations measured. Indeed, levels of anti-Xa activity as determined by a chromogenic assay were higher than expected. The trough level of anti-Xa activity measured 24 h after last drug administration (231 ng/ml) was in the range of expected Cmax levels (Groupe de travail RivaMoS Suisse, 2013). The level measured 4 days after last drug administration (34 ng/ml) was in the range of expected trough levels (Groupe de travail RivaMoS Suisse, 2013). Rivaroxaban plasma concentration measured by LC/MS-MS 48 h after administration (88 ng/ml) was comparable to approximately 8–16 h post-dose predicted levels based on a population pharmacokinetic model (20 mg q.d. administration) (Mueck et al., 2011). The plasma concentration measured 4 days after last drug administration (36 ng/ml) was comparable to expected trough concentrations. The estimated half-life was increased by threefold in our patient, approximately 24–30 h as compared to 11–13 h in the literature for old patients (Mueck et al., 2014).

Drug-Drug Interaction probably also played a role in rivaroxaban high levels. Crossover studies in healthy volunteers have shown significant increases in rivaroxaban systemic exposure during concomitant administration with strong inhibitors of CYP3A4/5 (and possibly CYP2J2), P-gp and BCRP. Steady state administration of ketoconazole led to a dose-dependent increase in rivaroxaban AUC and Cmax. AUC and Cmax increased by, respectively, 82% (90% CI 59%, 108%) and 53% (90% CI 27%, 85%) with ketoconazole 200 mg q.d. compared with rivaroxaban alone. With a higher dose of 400 mg q.d., the measured increases in AUC and Cmax were 158% (90% CI 136%, 182%) and 72% (90% CI 61%, 83%) (Mueck et al., 2013). As a consequence, the concomitant use of rivaroxaban with strong CYP3A4/5 and P-gp/BCRP inhibitors should be avoided due to a potential increase in the risk of bleeding (Mueck et al., 2013). According to the data from the ROCKET AF study (Prevention of Stroke and Embolism Trial in Atrial Fibrillation), the presence of combined CYP3A4/5 and P-gp inhibitors did not have any impact on safety outcomes such as bleeding events when comparing the rivaroxabanand warfarin groups (Piccini et al., 2016). However, key exclusion criteria included use of a strong CYP3A4/5 inhibitors or inducers. As recently underscored by Bouatou et al. (2016) this represents a major bias when analyzing the impact of polypharmacy pharmacokinetic interactions on the efficacy and safety of rivaroxaban. Moreover, the prescription of P-gp affecting drugs in heart failure patients is as frequent as 40–50% as shown by Jungbauer et al. (2010).

In the present case, our patient was receiving long-term treatment with simvastatin. Recently, an *in vitro* study has shown that simvastatin inhibited CYP3A4/5 and P-gp activity (Lee et al., 2015). An *in vivo* animal study performed by the same authors showed that concomitant administration of simvastatin with nifedipine, a CYP3A/5 and P-gp substrate, significantly increased the absolute bioavailability of nifedipine by 150% (Lee et al., 2015). On the other hand, atorvastatin inhibited CYP3A/5 but not P-gp activity *in vitro*, and had no impact on nifedipine pharmacokinetics *in vivo* (Lee et al., 2015). According to the FDA classification^1^, simvastatin would therefore be considered as a weak CYP3A/5 inhibitor (≥1.25 but <2-fold increase in AUC). CYP3A4/5 phenotyping, which was performed 8 days after the patient had been switched from simvastatin to atorvastatin, showed moderately decreased enzymatic activity as compared to our study population of healthy volunteers (Bosilkovska et al., 2014), which could be attributed at least in part to inhibition by atorvastatin. Although the enzymatic activity was only modestly decreased with a OH-midazolam/midazolam metabolic ratio of 0.31, compared to 0.50 in healthy volunteers without enzyme inhibitor/inducer, and 0.22 in the presence of a CYP inhibitor (voriconazole) (Bosilkovska et al., 2014), the measured phenotype allowed to rule out an ultrarapid metabolism.

The present case has some limitations. We did not investigate *ABCG2* and *CYP2J2* gene polymorphism. Indeed, CYP2J2 is also subject to genetic variations and the promoter region SNP (CYP2J2-76G>T; ^∗^7 allele) reportedly decreases epoxygenase activity *in vivo* (Murray, 2016). Moreover, we did not evaluate P-gp activity *in vivo* through phenotyping and the DDI with simvastatin has not been thoroughly investigated. Finally, the moderate acute renal failure at admission was probably a contributing factor to rivaroxaban high levels.

## Concluding Remarks

Our patient presented severe normocytic hypochromic anemia probably due to gastrointestinal bleeding, 3 months after switching his anticoagulant treatment from acenocoumarol to rivaroxaban.

Laboratory investigations showed high levels of anti-Xa activity and rivaroxaban plasma concentrations after rivaroxaban withdrawal, suggesting reduced rivaroxaban elimination (estimated half-life: 24–30 h). We suggest that the homozygous presence of *ABCB1* variant alleles and possibly altered CYP3A4/5 activity due to DDI with simvastatin were possible contributing factors, in addition to the moderate decreased renal function.

More clinical data are required to elucidate the impact of genetic polymorphism on rivaroxaban pharmacokinetics and bleeding complications. The impact of *ABCB1*, *ABCG2*, and *CYP2J2* gene polymorphism on rivaroxaban pharmacokinetics should be investigated in a phase 1 clinical study in healthy volunteers. The impact of genetic polymorphism on the susceptibility to DDI should also be further investigated. Indeed, the presence of a variant allele could predispose to DDI, as suggested between warfarin and simvastatin (Andersson et al., 2012).

## Author Contributions

KIL was in charge of the pharmacological investigations done in the patient, interpreted the results, and wrote the manuscript. YD measured the rivaroxaban plasma concentrations and performed the phenotyping test. PF was involved in the care of the patient and was in charge of the anti-Xa measurements and of interpretation of the results. JD supervised the investigations and the redaction of the manuscript. CS interpreted the results and wrote the manuscript. All authors read and approved the manuscript.

## Conflict of Interest Statement

The authors declare that the research was conducted in the absence of any commercial or financial relationships that could be construed as a potential conflict of interest.

## Footnotes

1 http://www.fda.gov/Drugs/DevelopmentApprovalProcess/DevelopmentResources/DrugInteractionsLabeling/ucm080499.htm
